# Moving the Agenda on Noncommunicable Diseases: Policy Implications of Mobile Phone Surveys in Low and Middle-Income Countries

**DOI:** 10.2196/jmir.7302

**Published:** 2017-05-05

**Authors:** George W Pariyo, Adaeze C Wosu, Dustin G Gibson, Alain B Labrique, Joseph Ali, Adnan A Hyder

**Affiliations:** ^1^ Johns Hopkins Bloomberg School of Public Health Department of International Health Baltimore, MD United States; ^2^ Johns Hopkins Bloomberg School of Public Health Department of Epidemiology Baltimore, MD United States; ^3^ Berman Institute of Bioethics Johns Hopkins University Baltimore, MD United States

**Keywords:** NCDs, policy, mHealth, policy analysis, surveys

## Abstract

The growing burden of noncommunicable diseases (NCDs), for example, cardiovascular diseases and chronic respiratory diseases, in low- and middle-income countries (LMICs) presents special challenges for policy makers, due to resource constraints and lack of timely data for decision-making. Concurrently, the increasing ubiquity of mobile phones in LMICs presents possibilities for rapid collection of population-based data to inform the policy process. The objective of this paper is to highlight potential benefits of mobile phone surveys (MPS) for developing, implementing, and evaluating NCD prevention and control policies. To achieve this aim, we first provide a brief overview of major global commitments to NCD prevention and control, and subsequently explore how countries can translate these commitments into policy action at the national level. Using the policy cycle as our frame of reference, we highlight potential benefits of MPS which include (1) potential cost-effectiveness of using MPS to inform NCD policy actions compared with using traditional household surveys; (2) timeliness of assessments to feed into policy and planning cycles; (3) tracking progress of interventions, hence assessment of reach, coverage, and distribution; (4) better targeting of interventions, for example, to high-risk groups; (5) timely course correction for suboptimal or non-effective interventions; (6) assessing fairness in financial contribution and financial risk protection for those affected by NCDs in the spirit of universal health coverage (UHC); and (7) monitoring progress in reducing catastrophic medical expenditure due to chronic health conditions in general, and NCDs in particular. We conclude that MPS have potential to become a powerful data collection tool to inform policies that address public health challenges such as NCDs. Additional forthcoming assessments of MPS in LMICs will inform opportunities to maximize this technology.

## Introduction

**Figure 1:**
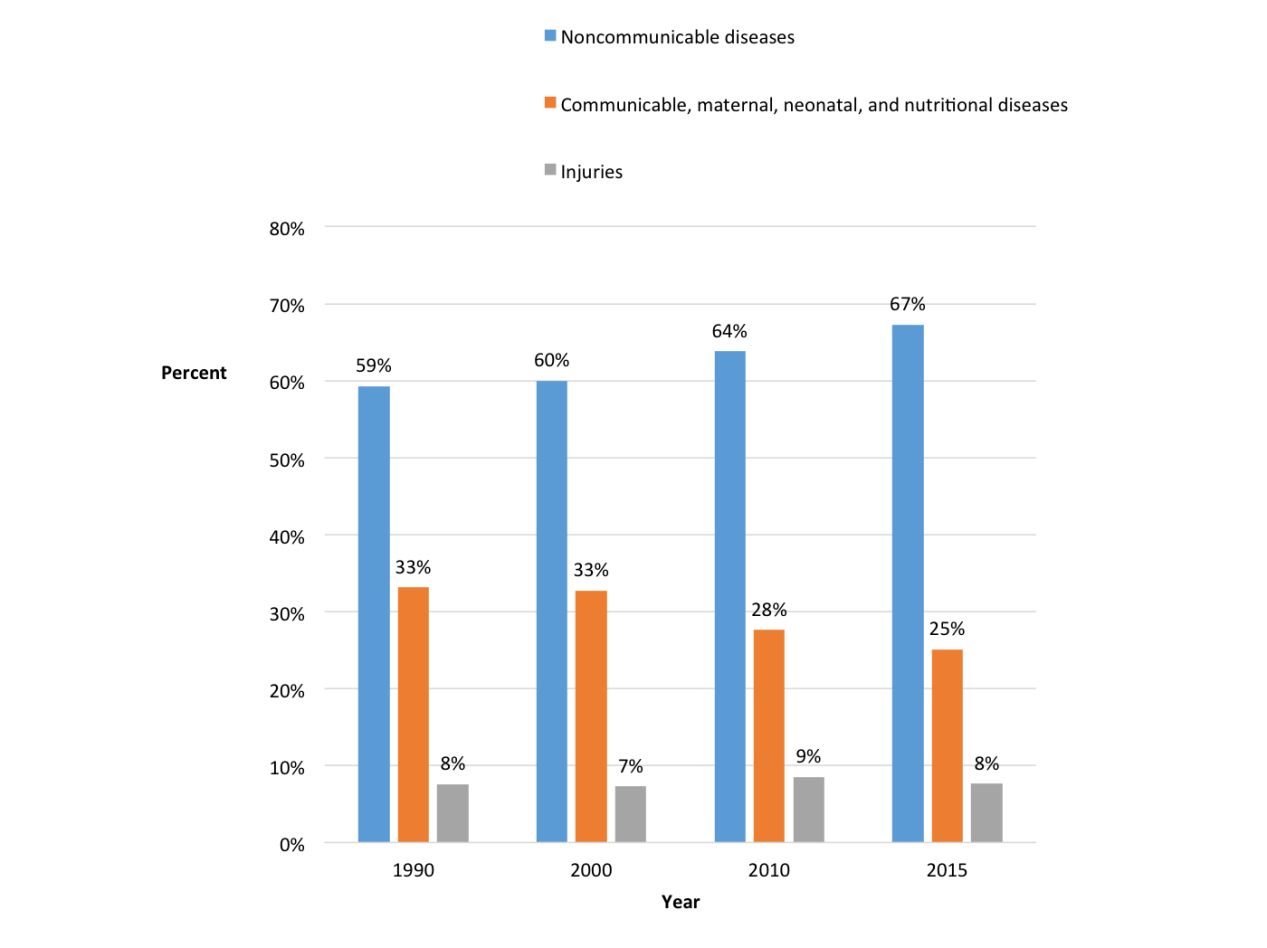
Changes in major causes of death in low- and middle-income countries (age-standardized): 1990-2015.

The growing burden of noncommunicable diseases (NCDs) such as hypertension, diabetes mellitus, obesity, asthma, and chronic obstructive pulmonary disease presents special challenges for health policy in low- and middle-income countries (LMICs) [1]. These challenges include priority setting and resource allocation in the context of resource constraints and lack of reliable and timely data for evidence-based policy and decision-making for NCD prevention and control. In LMICs, NCDs grew as a share of the major causes of death from 59% in 1990 to 64% in 2010 and were estimated at 67% in 2015 (calculations based on *Global Burden of Disease* report; see Figure 1) [1-2].

To assess the burden and prevalence of NCDs and risk factors in order to inform policy, programming, and to track progress, the World Health Organization (WHO) supports countries with the implementation and analysis of the STEPwise Approach to Surveillance (STEPS) of NCDs survey [3-4]. The STEPS survey contains 3 steps, or components, of NCD surveillance: (1) self-reported risk factor questionnaire, (2) physical measurement, and (3) biochemical measurement, all through face-to-face contact with respondents [4].

This paper highlights aspects of NCD-related health policy that decision-makers in LMICs face. These include identifying which risk factor-related priority interventions to implement, target groups to cover, how to ensure equity of coverage, and financial risk protection, among others. Decision-makers could benefit from newly available capabilities to conduct mobile phone surveys (MPS) in practically all settings, taking advantage of the growing ubiquity of mobile phones.

### Rising Mobile Phone Subscriptions Present an Opportunity

**Figure 2:**
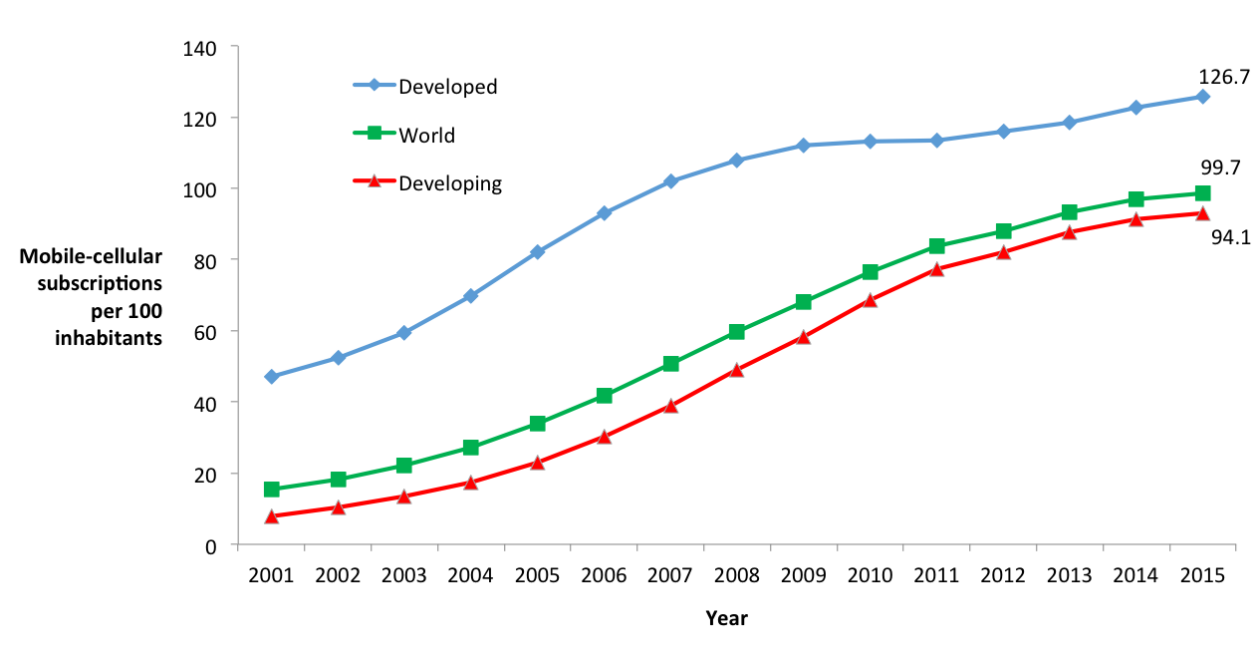
Mobile-cellular subscriptions per 100 inhabitants, 2001-2015.

To reduce the high costs and time requirements associated with conducting household surveys, higher income countries have developed and employed telephone and MPS to collect population-level estimates of health and demographics [5-6]. To date, such surveys are not widely used in LMICs. The global increase in mobile phone ownership and access has created an unprecedented opportunity to leverage mobile phones to revolutionize current methods of public health data collection in LMICs [6-9]; see Figure 2 [10]. Instead of relying only on face-to-face interviews conducted in respondents’ homes, it is now possible to conduct interviews on a range of public health topics remotely by delivering short surveys and interviewing respondents over their mobile phones. Options for survey modalities include the use of short message, service (SMS) or text message interactive voice response (IVR), and computer-assisted telephone interviews (CATI), collectively referred to as MPS.

### Global Commitments on NCDs

In 2011, the United Nations (UN) declared NCDs a public health emergency and called on member states to implement NCD prevention and control strategies. These include reducing the harmful use of alcohol, implementing the Framework Convention on Tobacco Control, promoting healthy lifestyles such as healthy diets (eg, rich in fruit and vegetables), increased physical activity, and legislation to control and reduce the sodium content of processed foods [11].

In 2012, the UN committed to achieving universal health coverage (UHC) through a resolution adopted by member states [12]; however, it will not be possible to achieve UHC without addressing challenges posed by NCDs. Through a resolution of the UN General Assembly in 2015, the UN member states adopted the sustainable development goals (SDGs) to replace the millennium development goals (MDGs) [13]. The third goal in the SDGs states “Ensure Healthy Lives and Promote Well-Being for All at All Ages” and calls for reducing premature mortality from NCDs through prevention, treatment, and promotion of mental health and well-being [13]. Noting an inadequate capacity for NCD surveillance, the WHO has called for such surveillance systems to be developed urgently and, where they exist, strengthened for monitoring NCDs, and their risk factors [4,14]. Innovative solutions are needed to strengthen national NCD policies and their implementation, monitoring, and evaluation, and to ensure equity [15].

The WHO has developed an NCD global action plan with six objectives to (1) raise the priority accorded to prevention and control, (2) strengthen national capacity, leadership, governance, multisectoral action, and partnerships for prevention and control, (3) reduce modifiable risk factors and underlying social determinants, (4) strengthen and orient health systems to address prevention and control, and underlying social determinants, (5) promote and support national capacity for high-quality research and development for the prevention and control, and (6) monitor the trends and determinants of NCDs and evaluate progress in their prevention and control [14]. The basis for monitoring progress on implementation of the global action plan is the WHO NCD monitoring framework which has 25 indicators, and proposes a set of 9 voluntary global targets that member states can choose to track and report on an annual basis [3].

### Getting NCDs on the National Policy Agenda

Having signed onto global commitments for UHC and the SDGs, countries have to find practical ways to translate these commitments into action. A comprehensive policy and practice framework for NCD surveillance, prevention, and care is essential to meeting national commitments to UHC and SDGs. Countries need to assess how and on what basis NCD policies are being formulated. Studies in a number of LMICs show that NCDs are a growing burden while health services are still largely inadequate to meet the needs of the population [16-18].

Translating commitments into action involves governments exercising stewardship and governance in the health system. One of the primary goals of health systems is to improve population health [19]. Currently, there exists limited good quality evidence on the state of NCD risk factors and effectiveness of NCD prevention and control efforts in LMICs [14,20]. Increasingly, countries are developing national policies and action plans for NCD prevention and control. For instance, the WHO reports that in 2014, of a total of 194 member states, 160 (82%) had regulations on age limits for the sale of alcohol, 76 (39%) had a written national policy on alcohol, and 52 (27%) had taken steps to implement such policies. In 2013, 95 (49%) of WHO member states had implemented at least one of four key tobacco control interventions [21].

Apart from countries that have implemented STEPs surveys and have population-based data on NCDs, many LMICs still largely rely on data collected at health facilities. Such data are inadequate given numerous limitations in LMIC settings including incompleteness, inaccuracies, poor quality, and lack of resources for reliable data storage, retrieval, analysis, and reporting [22,23]. Gathering data on population-based risk factors and existing NCD-related behaviors is greatly needed.

**Figure 3:**
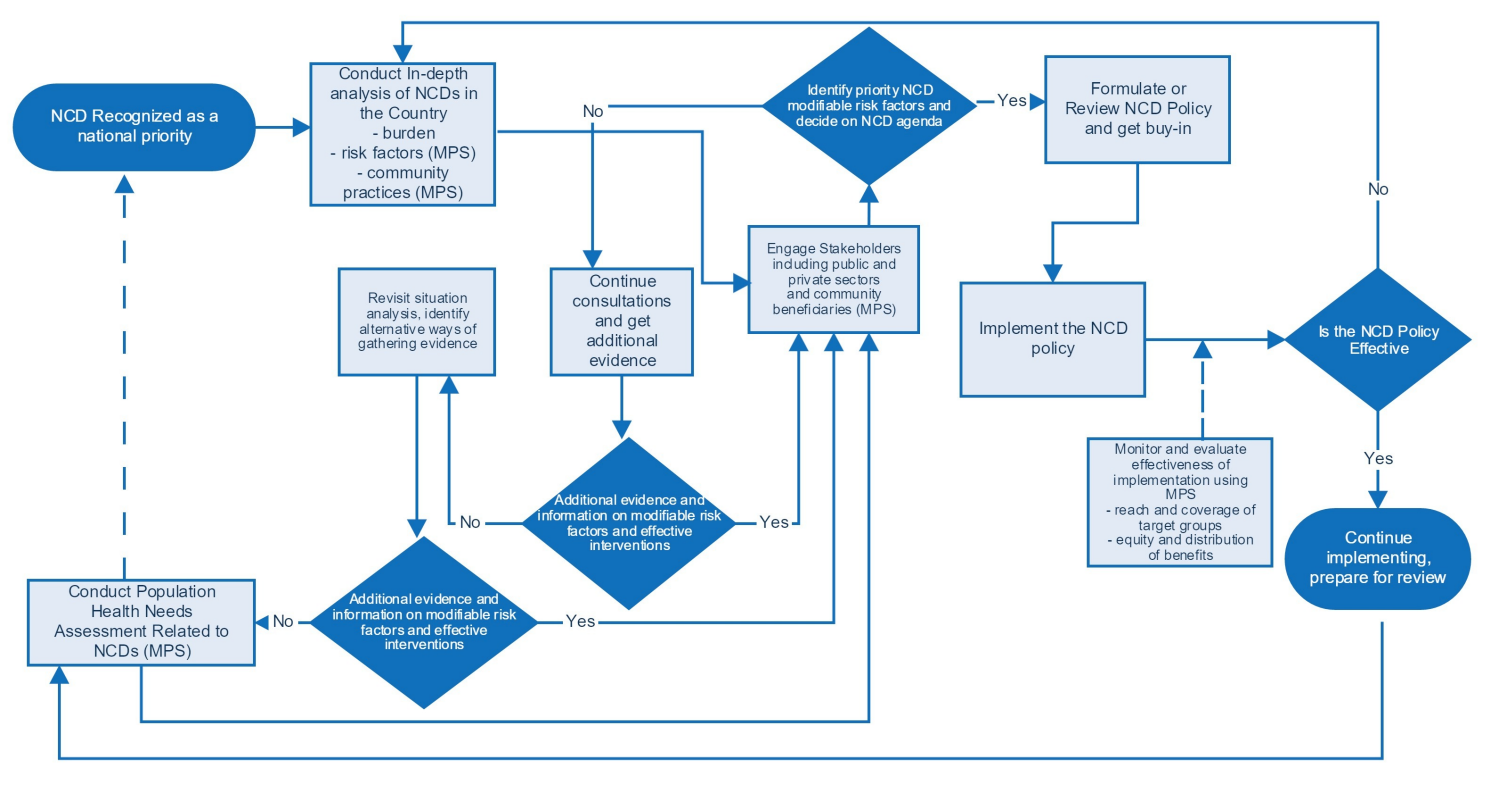
Pathways for mobile phone surveys to influence noncommunicable disease policy.

According to the paradigm of punctuated equilibrium periods of stasis in public policy are shaken from time to time by innovations or political events, such as change of government, which presents a window of opportunity and accords new priority to an issue or changes the dynamics [24,25]. Following years of relatively limited action, the MPS innovation represents a potential game-changer for national NCD policy-making in LMICs. MPS provides an opportunity for strengthening national agenda setting [24,25], including taking evidence-based action in the areas of NCD prevention and control. The technology can help generate new information rapidly, which can indicate the effectiveness or lack of a national policy, and thereby help to challenge the status quo of equilibrium [25,26]. The pathways through which MPS may influence NCD policy are highlighted in Figure 3.

Once NCDs are recognized as a current or emerging priority, a country may commission a situation analysis that would provide an in-depth assessment of the NCD burden, risk factors, and existing policy and programmatic responses. Surveys conducted using mobile phones could potentially play a role in generating population level evidence particularly as it pertains to behavioral risk factors and service utilization. Such an analysis would be useful in engaging key stakeholders from the public and private sectors, professional groups, civil society, academics and researchers, and health development partners. Such engagement could result in a clearer identification of the modifiable risk factors in an agenda setting decision-making. The policy formulated in this process has to be implemented. Again MPS could play a key role in monitoring population level reach, acceptability, and effectiveness of interventions. Information collected rapidly from MPS during implementation would help inform monitoring of progress as well as contribute to program evaluation. These activities would provide new input into the identification of population level needs and a repetition of the policy cycle.

In using MPS-generated data for decision-making, it should be kept in mind that no tool or approach is without shortcomings. MPS are not free of bias. The information collected from target populations will be a representative of the population that owns mobile phones [27]. It is also still to be explored how the findings from MPS can be interpreted, taking into account people likely to be unreached or underrepresented, such as people who do not own mobile phones or who live in areas where network coverage is patchy or nonexistent. A number of research and development aspects related to the use of MPS still need to be adequately addressed [28]. Similarly, a number of MPS development challenges such as increasing survey completeness, representativeness and response rates, as well as the potential role for use of incentives are to be kept in mind and are discussed elsewhere [29].

### Identifying Priority NCD Modifiable Risk Factors

Strategies to collect population level NCD-related risk factor data through face-to-face household surveys, such as the WHO STEPs surveys, demand time and financial resources even though they provide a comprehensive picture every 5-7 years. Although MPS are not a substitute for current surveys administered through face-to-face interactions, they offer promise as interim and rapid assessment tools. They can be useful in identifying population level changes in modifiable risk factors such as physical activity levels, dietary intake, smoking, and harmful use of alcohol; and help inform equitable coverage of interventions such as NCD-related health messaging or access to care.

MPS can assist policy makers in identifying high-risk groups, such as groups with above-average prevalence or clustering of behavioral risk factors, for better targeting of interventions. During priority setting, the community—a key stakeholder—often is difficult to engage to get their perceptions and inputs [30-33]. With MPS, this barrier can potentially be overcome, for example, by polling people at the population level on their policy or resource allocation preferences, or the extent to which particular services are reaching them. As shown in other sectors, with MPS, the time for gathering population-based data, as well as time for analysis and data sharing can be considerably shortened compared with traditional approaches of data collection. For example, the use of mobile phones for rapidly collecting data to inform policies has been documented by the World Bank-supported *Listening to Africa* (L2A) and *Listening to Latin America and the Caribbean* (LAC) programs, as well as others. The L2A and LAC are pilot programs that recruit panels of respondents from the community level, and upon obtaining consent, call them regularly to collect community views and experiences on a wide range of topics including provision of services such as health, education, water and sanitation, and emergency assistance following a crisis. For instance, in Tanzania, the World Bank partners with Twaweza, a local nongovernmental organization. Twaweza’s “sauti za wananchi,” Swahili for “voices of citizens,” collected data monthly from a representative population sample on topics such as education, health, a new tax, and the new national constitution [8,9,34,35]. These experiences suggest that, although not yet widely used in public health policy, there is strong potential for wider use for improved citizen engagement in the public policy space, which would benefit public health.

Schmidt and Barnhill [15] argue that, in order to achieve the SDGs, it will be important to take into account equity dimensions in implementing interventions for NCDs. The concern for improving health equitably is central to public health policies. MPS offer a possibility to generate timely population-based data at regular intervals thus permitting a review of existing NCD priorities, identifying which groups are being reached or not reached, and taking into account community perspectives.

### Engaging Stakeholders in NCD Policy Formulation

Public health policy formulation and analyses have traditionally tended to be dominated by technocrats. Policy beneficiaries, while one of the key groups of actors in the policy triangle made up of content, context, and process [36], are rarely, if ever, consulted on their real needs, perceptions, preferences, and utilization of existing facilities. Even if an intervention is technically sound, lack of involvement of intended beneficiaries may result in formulating a less appropriate policy and decrease its effectiveness along the coverage continuum [37,38]. However, providing information such as may be collected from MPS is a necessary though not sufficient input into the process. Engaging communities also requires an enabling environment and democratic culture of consultation and public dialog, which go beyond the realm of public health, and have implications on governance and the stewardship role of government on one hand as well as strong civil society on the other. Success in formulating an adequate public health policy and implementing it depends on concerted action at various levels, involving partnerships in the public and private sectors, community representatives, political leaders, technical experts such as researchers and academics, and representatives of professional groups [36]. Beneficiary communities are not simply passive recipients of policy interventions; they are among the key actors in the uptake of policy interventions. Their acceptance or rejection of policies can make the difference between success and failure. Using MPS to identify or track trends in key NCD risk factors can be a catalyst to initiate engagement or strengthen the involvement of various stakeholders in order to more adequately address the NCD challenge. Identifying a trend showing increases in binge drinking through rapid surveys on harmful use of alcohol among teenagers, for instance, could act as a stimulus to engage with youth groups, parents, educators, political leaders, health professionals, and behavior change advocates to identify the most effective messaging and strategies to address the issue.

There is potential for MPS to provide timely new information that leads to building a coalition of support during the process of NCD policy reviews [36]. New actors or policy entrepreneur [39] could emerge from any of the stakeholder groups and help strengthen the NCD policy development process. In this way, beneficiaries become central actors rather than passive bystanders, in a way that is consistent with global movements toward patient- and client-centered health management. Although this may demand new skills to incorporate such data in meaningful ways, potential exists to facilitate better planning and delivery of interventions to mitigate NCD risk factors. The negotiation between what is technically feasible and politically and practically realistic is an essential aspect of policy development and implementation [40,41].

### NCD Policy Implementation and Monitoring

Successful policy implementation needs more than a technically sound intervention. Frieden [42] proposes the following six components that are essential for effective public health program implementation: (1) innovation to develop evidence base for action; (2) technical package of a limited number of evidence-based interventions that will have a large impact; (3) managing performance, especially through rigorous real-time monitoring and evaluation leading to improvement in program implementation; (4) partnerships and coalitions (eg, between public and private sectors); (5) communication of accurate timely information to decision-makers, implementers, and the public; and (6) political commitment to obtain resources and support for effective action. MPS can contribute in generating timely evidence and speeding up the availability of information to various actors in the policy implementation process. Implementation of policy in real life is also influenced by perceptions of a situation during the interface between implementers and beneficiaries. The final step in the implementation chain is dependent on the interpretations of the policy by those functionaries at the interface with users. Lipsky [43] called these final implementers as street-level bureaucrats and posits that this effectively makes them policy makers. The implementers, for example, nurses in a clinic, or community health workers (CHWs) in a village, have the ability to influence perceptions of the users and communities of the intended policy beneficiaries. The potential to regularly assess the coverage, frequency, and effectiveness of the interface with beneficiaries during implementation using MPS gives the policy maker higher up in the system an unprecedented means to gauge what is happening on the ground in reality (Table 1). This can be particularly useful in informing policy makers in LMICs on what services are being offered, who is delivering them, and how national level policies are received. This is important especially as these countries tend to have a mix of formal public and private sector providers as well as a significant role played by other informal actors including quacks and traditional and complementary medicine practitioners. The use of MPS can help flag emerging needs and opportunities for course correction before too much time has passed and potentially avoid unnecessary wastage of scarce resources on ineffective interventions.

Policy makers can use MPS to monitor (1) whether services are reaching intended beneficiaries or not and (2) other important aspects as shown in Table 1. With rapid monitoring, ineffective interventions or groups not being reached with current levels of effort and resource allocation can be detected early in the course of policy implementation. Policies often have unintended and unwanted consequences [36], the effects of which often become known after a long period of implementation and after considerable resources have been deployed. MPS can help to rapidly identify unintended or unwanted consequences so that the managers can take remedial action. In Malawi, for example, feedback from communities was used by health care managers in deciding to change the mix of personnel delivering services through a task sharing arrangement [17]. In South Africa, CHWs used mobile phones to increase screening for cardiovascular diseases at the community level, effectively increasing screening rates and reducing errors [44]. MPS can help to rapidly collect data on how much people are spending on NCD-related health care costs and monitor for the risk of catastrophic NCD-related health expenditures, especially given that many people are outside the health care financial safety system, even in the era of UHC, particularly in LMICs. Htet et al [45] found that, in Myanmar, up to 60% of out-of-pocket expenditure for NCDs was for medicines. For personnel, delivering care such as CHWs, MPS can help empower them with access to rapid survey-based information to target their activities. Mayosi [46] makes the case for the need to integrate surveillance and care of NCDs.

### Evaluating NCD Policy Effectiveness

Finally, every policy should be evaluated to assess its effectiveness, revise the policy, or change direction altogether. Sabatier [26] estimated that typically one needs up to a decade for a reasonable evaluation of a policy’s impact. Part of the long lead-time is because policies need time to take root and be effectively implemented. Some of the delay, however, is due to the time taken to implement, analyze, and report findings from surveys using traditional methods. For some of the delay due to long lead-time of traditional surveys, MPS have the potential to shorten the cycle by making information more frequently available for use in monitoring trends and for evaluation of policy effectiveness and impact. Managers, for instance, can assess service coverage using a continuum to include (1) availability: which services are available to the community, (2) accessibility: how accessible are the services, (3) acceptability of services, (4) actual utilization, and (5) effectiveness of utilization [38]. Such assessments would help inform policy and programs, for example, extent of catastrophic expenditure arising from NCD-related health care and its causes may lead to resource re-allocations to better address population needs.

Table 1 summarizes some of the main topics relevant to NCD policy formulation, monitoring implementation, and policy evaluation, and the potential role of using MPS to generate the information needed.

**Table 1:** Examples of ways mobile phone surveys can contribute to noncommunicable disease (NCD) policy formulation, monitoring implementation, and evaluation (based on authors’ assessments and expectations).

Topic or issue	Examples of information needed for policy and program management	Potential contribution from using MPS^a^ (high, medium, low)		
		Formulation	Monitoring	Evaluation
Service delivery and utilization	Services most needed at the community level; groups most affected by different risk factors; where people seek care for NCD^b^-related services; frequency of contact between providers and users	High	High	High
Equity	Whether service delivery is equitable; who is being reached with interventions or not?	High	High	High
NCD care benefit packages	Informing selection, for example, priority target groups to benefit from NCD-related services and financial subsidies; tracking achievements of targets; assessing household care utilization; and economic implications	Low	Medium	High
Public-private partnership	NCD-related services being accessed through the public or private sector; effectiveness of contracted providers in reaching beneficiaries	High	Medium	High
Continuity of care	Coverage of continued care in the community, for example, people with hypertension who have their blood pressure monitored close to where they live	Low	High	High
Access to essential medications	Access to medications close to where people live	High	High	High
Behavior change efforts	Source and uptake of behavior change communication messages; role of incentives and disincentives to facilitate healthy behavior, for example, increasing physical activity	Medium	High	High
Fairness in financial contribution and financial risk protection	Public spending and subsidies aimed at the poor—if reaching the intended beneficiaries and preventing catastrophic medical expenditure; costs of seeking care for NCD-related conditions and source of payments	Low	High	High
Health system responsiveness	Whether services are responsive to people’s expectations, user-satisfaction with existing NCD services	Medium	High	High
Health management information systems	Triangulating data from routine facility-based information systems with population-level data, for example, on characteristics of service users	Low	Medium	High
Universal health coverage	Coverage of the population in scope and reach of NCD^b^ services	Low	High	Low
Pharmaceutical policies related to NCDs	Drugs to allow for use at community level; how to monitor safe use; rational drug use	Low	Medium	High

^a^MPS: mobile phone surveys.

^b^NCD: noncommunicable disease.

## Discussion

NCDs present special challenges for health policy, especially in LMIC settings. How can recent advances in mobile phone technology (eg, ability to conduct national surveys more frequently than traditional public health surveys and at lower cost), be leveraged to strengthen formulation, implementation, and evaluation of health policy? The coming together of problems, alternative solutions, and politics can create windows of opportunity for action [25]. We advocate for going beyond current efforts of collecting NCD-related data. Current efforts are based on passive identification of persons already affected by NCDs who seek care at health facilities and costly population-based household surveys. MPS can help to speed-up population-based risk factor surveillance, monitoring of trends, identification of high-risk groups, and monitoring perceptions and effectiveness of interventions at population level more frequently than existing face-to-face surveys. The high costs and time commitments of conducting face-to-face surveys on NCD risk factors means that they are conducted infrequently, about every 5-7 years. Although they generate data that are useful for long-term planning and evaluations, these surveys are costly and take time to plan, fund, and conduct. Consequently, national policy makers and program managers, who wish to track progress and do course correction in a more timely fashion, are left with a gap. With scientifically sound methodology and robustness, MPS potentially provide a more cost-efficient and timely mechanism to collect data on NCD risk factors and other public health priorities.

It is our hope that the near future will see an increase in studies that examine the role of MPS in health in LMICs. A review by Peiris [47] found that only a few studies have examined the role and potential for mHealth to improve health, especially in LMICs. Bloomfield et al [48] found limited evidence on use or benefit for health of mHealth in sub-Saharan Africa, despite the growing use of mobile devices. Such evidence points to mHealth being an underexploited resource [48]. During the process of scanning for policy alternatives, MPS can help to zoom in on those requiring a closer look and thus promote more relevant options [49].

The potential role of public-private partnerships and ethical, legal, and societal issues that arise in using MPS to serve the public good are still to be investigated [50]. Furthermore, MPS can at best serve as a complement to household surveys—face-to-face interactions are still needed for obtaining measurements such as body weight and height, and blood samples for glucose and lipid tests, at least for the foreseeable future.

Despite some limitations, MPS can facilitate engaging communities to better understand their perspectives, concerns, preferences for service delivery, and continuity of care, in order to develop policy options that are accessible to communities, for example, a drug policy that ensures people access appropriate medications for NCDs close to where they live and establishing public-private partnerships with drug shops and clinic operators.

The issues we have highlighted in this paper are neither comprehensive nor exhaustive. There are certainly other key gaps in knowledge that will be relevant for formulation or implementation of policies on NCDs relevant for prevention, risk factor surveillance, treatment and control, and rehabilitation. We have tried to focus on those which, in our view, are amenable to being informed by MPS.

It is imperative that prevention strategies be developed which address NCDs and monitor trends of risk factors [4,51]. In this paper, we have attempted to argue that MPS represent a disruptive innovation [52] that can help in this endeavor taking advantage of a convergence of opportunities. The recognition of NCDs as a global public health emergency, availability of interventions, and improved capabilities to generate timely information using MPS represent emerging multiple streams [25]. The time has come to take advantage of these to benefit NCD prevention and control policy.

### Conclusions

MPS offer a powerful tool for collecting population-based data that can help inform policies to address key public health challenges such as NCDs. MPS can be developed for use at national and subnational levels. This paper has laid out some of the key NCD-related policy areas and processes that could benefit from the emerging possibility of high-quality MPS in LMICs. More forthcoming assessments of MPS in LMICs will help to provide real-world experiences.

If LMICs are going to deal effectively with the existing and growing burden of NCDs, they cannot afford to continue using a passive approach of waiting for people to fall sick with NCDs and then attempting to treat. Furthermore, MPS have potential to promote improved accountability and transparency in policy processes through regularly sharing results based on the most current population-based data, which will be increasingly important in the context of global attention on NCDs and national efforts in the push for UHC.

## Abbreviations

CATI: computer-assisted telephone interviews
CHW: community health worker
IVR: interactive voice response
LAC: Latin America and the Caribbean
L2A: Listening to Africa
LMICs: low- and middle-income countries
MDGs: millennium development goals
mHealth: mobile health
MPS: mobile phone survey
NCD: noncommunicable diseases
SDGs: sustainable development goals
SMS: short message service
STEPs: WHO STEPwise Approach to Surveillance of Noncommunicable Diseases
UHC: universal health coverage
UN: United Nations
WHO: World Health Organization
